# Mild Sensory Stimulation Completely Protects the Adult Rodent Cortex from Ischemic Stroke

**DOI:** 10.1371/journal.pone.0011270

**Published:** 2010-06-23

**Authors:** Christopher C. Lay, Melissa F. Davis, Cynthia H. Chen-Bee, Ron D. Frostig

**Affiliations:** 1 Department of Neurobiology and Behavior, University of California Irvine, Irvine, California, United States of America; 2 Center for the Neurobiology of Learning and Memory, University of California Irvine, Irvine, California, United States of America; 3 Department of Biomedical Engineering, University of California Irvine, Irvine, California, United States of America; Julius-Maximilians-Universität Würzburg, Germany

## Abstract

Despite progress in reducing ischemic stroke damage, complete protection remains elusive. Here we demonstrate that, after permanent occlusion of a major cortical artery (middle cerebral artery; MCA), single whisker stimulation can induce complete protection of the adult rat cortex, but only if administered within a critical time window. Animals that receive early treatment are histologically and behaviorally equivalent to healthy controls and have normal neuronal function. Protection of the cortex clearly requires reperfusion to the ischemic area despite permanent occlusion. Using blood flow imaging and other techniques we found evidence of reversed blood flow into MCA branches from an alternate arterial source via collateral vessels (inter-arterial connections), a potential mechanism for reperfusion. These findings suggest that the cortex is capable of extensive blood flow reorganization and more importantly that mild sensory stimulation can provide complete protection from impending stroke given early intervention. Such non-invasive, non-pharmacological intervention has clear translational potential.

## Introduction

Over the last few decades, adult cortical plasticity has been increasingly recognized as a fundamental mechanism underlying a host of brain processes involving synapses, neurons, neuronal circuits, representational maps, and supporting metabolic and vascular systems [1]. Plasticity is commonly taken advantage of in rehabilitation therapy after brain damage such as that caused by ischemic stroke [2]. More direct methods of inducing plasticity, such as electrical stimulation of the cortex, have also demonstrated their capacity to improve recovery after ischemic injury [3]. Despite great advances in such plasticity based therapies, however, stroke remains the number one cause for long term disability and the third leading cause of death in the United States [4]. Could cortical plasticity be exploited not only in post-injury rehabilitation, but also to protect the cortex from an impending ischemic injury? Because our laboratory has previously demonstrated that whisker stimulation can induce extensive plasticity in cortical whisker functional representations [5], [6], [7], we were led to consider whether the interactions between activated cortical representations of whiskers and their supporting cortical vasculature are also plastic, and whether such plasticity could be exploited to provide protection from impending stroke.

To investigate this question, we tested whether non-invasive sensory stimulation (intermittent mechanical stimulation of a single whisker) could confer protection from a permanent middle cerebral artery occlusion (pMCAO) in a rat model of ischemic stroke. The permanent MCAO was achieved via a double ligature and transection which resulted in a reduction in local levels of oxyhemoglobin, total hemoglobin, and oxygen saturation, and caused substantial infarct [8]. The pMCAO ischemic stroke model described was chosen because middle cerebral artery (MCA) is the most common location for stroke in humans and ischemic stroke is the most common type [4]. Also, because MCA supplies blood to the barrel cortex, a portion of the somatosensory cortex which is highly functionally and anatomically ordered, this model allows ischemic conditions to be created in an area where evoked cortical responses can be induced and assessed, and changes clearly measured.

Here we describe complete protection from impending stroke when single whisker stimulation was administered within a one (and often two) hour time-window following ischemic onset, as confirmed by functional imaging, neuronal recording, histological analysis, and behavioral assessment. Animals that did not receive this whisker stimulation until 3 hours after ischemic onset lost functional response, sustained extensive cortical infarct, and demonstrated impaired behavior typical to animals that have sustained stroke. We also report blood flow data, collected 24 hours post-occlusion, suggesting that whisker stimulation delivered within the protective time window results in the redirection of blood flow into the occluded MCA from an alternate arterial source via collateral vessels. Thus reversed flow in the occluded MCA is a likely source of reperfusion of the ischemic area and protection from ischemic damage.

Our results suggest that mild sensory stimulation is capable of inducing plasticity sufficient to save the cortex from impending stroke if administered within a critical time window. These findings represent a new direction in research concerning the brain's innate capacity for therapeutic plasticity.

## Results

**Figure 1:**
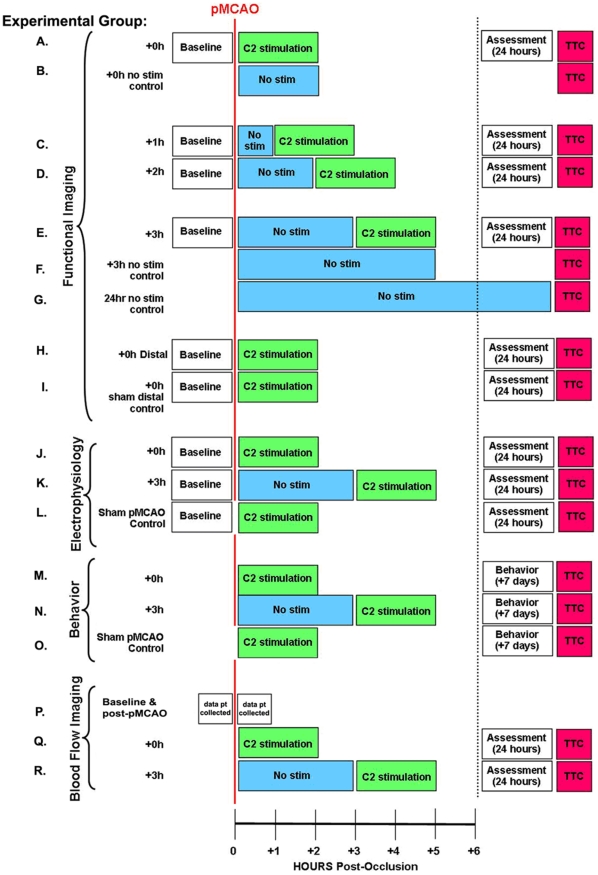
Schematic of the Experimental Design with Constituent Experimental and Control Groups. For all groups (except sham pMCAO controls that never undergo MCA occlusion) the red vertical line indicates time of permanent occlusion of the MCA (pMCAO), the x-axis at bottom demarcates the chronology of events post-occlusion, and the black dotted line indicates the initiation of all second day experimentation. (Note, however, that the behavior animals are examined for the first time at one week post-pMCAO). (A–L) Experimental paradigms and chronology of events are listed for animals undergoing either functional imaging or electrophysiology (M–O). Chronology of events for animals undergoing behavioral assessment one week post-occlusion (P-R). Experimental paradigms and chronology of events are listed for animals undergoing blood flow imaging. N-sizes for each treatment condition are as follows: (A) n = 7; (B) n = 3; (C) n = 7; (D) n = 8; (E) n = 7; (F) n = 5; (G) n = 2; (H) n = 3; (I) n = 3; (J) n = 3; (K) n = 3;(L) n = 21 (M) n = 22; (N) n = 22; (O) n = 22; (P) n = 13; (Q) n = 6;(R) n = 6.

Our experimental groups consisted of animals who received pMCAO and whisker stimulation, with the different groups varying only in the time delay between the occlusion and the initiation of stimulation. Regardless of experimental group, all animals were sedated for an equivalent amount of time (group mean±SEM, hours under anesthesia: no-stimulus pMCAO: 10.8±1.9, +0 h: 9±0.5, +1 h: 9.64±1.6, +2 h: 10.3±0.5, +3 h: 10.9±0.5, Distal occlusion subjects: 9.56±1.7, sham pMCAO: 9.35±0.6 h; F_6,143_ = 1.09, p = 0.37, ANOVA). Subsets of each of these groups were assessed using one of several techniques. All experiments and their controls are outlined in Figure 1. All of these animals were assessed for infarct histologically.

### Whisker stimulation can protect the adult rat cortex from structural, functional, and behavioural impairment following pMCAO

#### Healthy cortical tissue was maintained if subjects received whisker stimulation immediately after pMCAO, but not if stimulation was delayed for 3 hours

**Figure 2:**
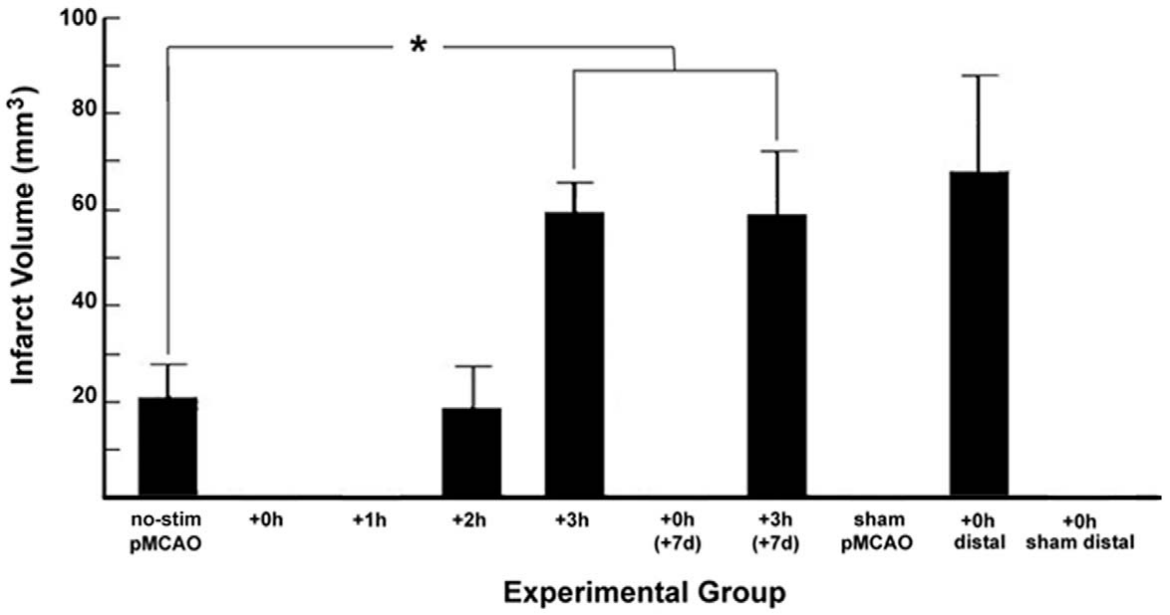
Experimental Infarct Volumes. The total volume of infarct tissue sustained by animals which underwent: pMCAO (no-stimulation control animals, n = 10), pMCAO and immediate whisker stimulation (+0 h, n = 38), or pMCAO and whisker stimulation initiated three hour post-occlusion (+3 h, n = 38) as assessed via 2,3,5-Triphenyltetrazolium Chloride (TTC) assay for infarct. No-stimulation control animals never received whisker stimulation, yet underwent identical anesthetic and surgical procedures (including pMCAO) as +0 h and +3 h groups, were only assessed using TTC. Smaller subdivisions of the larger experimental +0 h and +3 h groups were assessed using various other techniques as fully described in the remainder of this article. Asterisk indicates a significant difference in infarct volume between the no-stimulation controls and +3 h subjects. Note that multiple groups did not sustain infarct are included here for the sake of comparison.

In this study, the presence of ischemic infarct was determined using a 2,3,5-triphenyl tetrazolium chloride [TTC] solution which is widely used to evaluate infarct in neural tissue following experimental ischemia [9], [10], [11]. Infarct volumes measured with TTC correspond closely with those measured using other histological techniques [9], [12], [13] and is commonly used successfully in the assessment of neuroprotective agents in cerebral ischemia studies [9], [10], [11]. Control animals that underwent pMCAO and experimental animals were sedated for an identical amount of time, but control animals never received whisker stimulation. The control animals sustained large cortical infarctions ranging in volume from 13.9–35.0 mm^3^ (no-stimulation controls, n = 10, mean = 28.4±2.4 mm^3^, Figure 2). In contrast, animals that received whisker stimulation immediately following pMCAO (+0 h, n = 38, Figure 2) did not sustain any infarct. When whisker stimulation was delivered three hours after pMCAO (+3 h, n = 38, Figure 2), however, infarct volumes were even greater than those observed in no-stimulation control animals (range = 47.6–87.3 mm^3^, mean = 61.4±2.4 mm^3^; Figure 2; Mann-Whitney *U* = 367, *n*_1_ = 10, *n*_2_ = 38, p = 7×10^−6^). These data suggest that while whisker stimulation is highly protective when delivered immediately post-occlusion, when delivered three hours after pMCAO, it not only fails to provide protection, but actually exacerbates the ischemic pathology.

#### Whisker functional representation was maintained if subjects received whisker stimulation immediately after pMCAO, but not if stimulation was delayed for 3 hours

**Figure 3:**
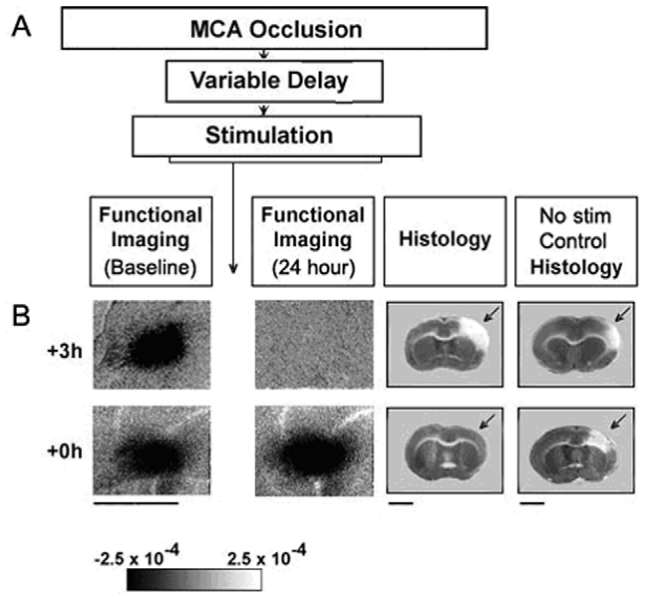
+0 h animals maintained whisker functional representation and sustained no infarct. A: Schematic of the ISOI experiments. The vertical arrow denotes manipulations which were performed between the two functional imaging sessions. B, left half: +3 h and +0 h subjects' images of the ipsi-ischemic C2 whisker functional representation collected before and 24 hours after pMCAO. Scale bar indicates 5 mm. Linear grayscale bar indicates intrinsic signal strength ×10^−4^. Black and white streaks correspond to large surface blood vessels. B, right half: Representative coronal sections taken from experimental and control animal's TTC assay for infarct. Note that the area devoid of staining (arrows) within the +3 h subject's and control subject's left cortex indicate ischemic infarct due to an occlusion of the left MCA. +0 h no stimulation controls and +3 h no stimulation control rats were anesthetized for the same duration as their respective experimental group and underwent the same pMCAO surgical procedure, but never received whisker stimulation. Scale bar indicates 5 mm.

**Figure 4:**
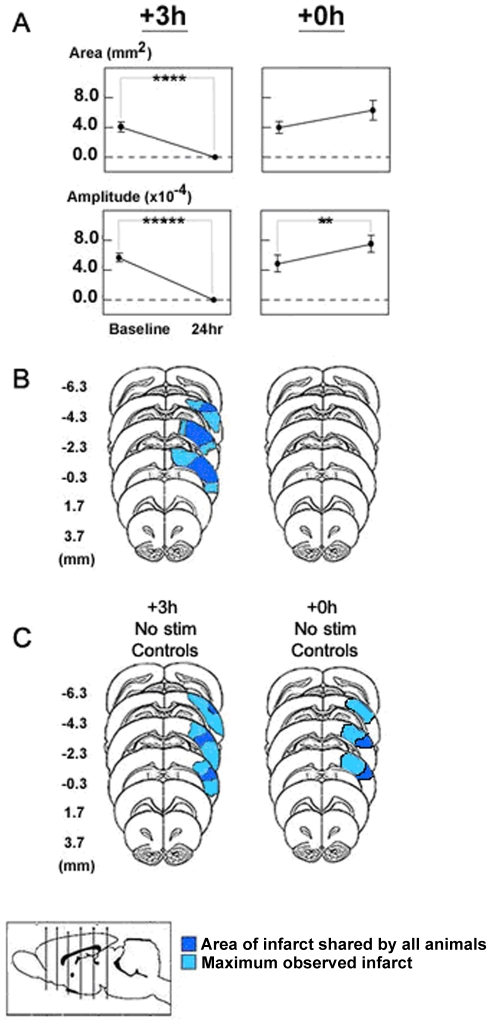
+0 h animals maintained baseline level (or stronger) whisker representation and sustained no infarct. A: In each plot, group baseline is paired with +24 hour data. Means and standard errors are provided for the area (first row) and amplitude (second row) of the ipsi-ischemic C2 whisker functional representation before and 24 hours after pMCAO. A value of zero indicates no response to whisker stimulation. Asterisks indicate significant differences between baseline and 24 hour values (** p = 0.0086, **** p = 0.00082, ***** p = 0.000001). B, C: Location and extent of infarct due to ischemia present in the +3h and +0h experimental groups (B), and +3h and +0h No-stimulation controls (C) according to TTC analysis. Color coding indicates area of infarct common to all subjects (dark blue), and maximum observed infarct (medium blue). Atlas legend includes six vertical lines which correspond to each coronal section at 2 mm intervals from bregma. Brain map adapted from [52].

In order to assess cortical function before and after pMCAO, we used the functional imaging technique Intrinsic Signal optical imaging (ISOI), which has been used extensively to provide high spatial resolution maps of stimulus evoked hemodynamic-related signals as an indirect means to image the functional organization of the cortex, and examine how these signals contribute to brain function [14], [15], [16] (for a recent review see Frostig and Chen-Bee, 2009 [17]). ISOI revealed that the C2 whisker functional representation in the ischemic cortex (referred to hereafter as ipsi-ischemic C2 representation) had disappeared completely in +3 h rats at 24 hours post-pMCAO (n = 7, Figure 1B). Its absence was confirmed using a threshold five times lower than that used for routine quantification (see Figure S1). Thus, whisker stimulation failed to provide structural or functional protection from ischemic injury when delivered three hours after pMCAO. In contrast, all rats that received whisker stimulation immediately (+0 h, n = 7, Figure 1A) after pMCAO maintained an ipsi-ischemic C2 representation equivalent to or greater than baseline representation at 24 hours post-pMCAO (Figure 3 and 4). These data suggest that pMCAO alone was effective in creating ischemic conditions (as evidenced by the control animals) and that whisker stimulation is capable of protecting the cortex from this ischemia. In summary, if whisker stimulation was delayed for three hours after pMCAO, the ipsi-ischemic C2 functional representation disappeared and the corresponding cortex sustained substantial infarct, whereas if the identical whisker stimulation was delivered immediately after occlusion, subjects maintained pre-pMCAO baseline level C2 functional representations and sustained no infarct.

We then assessed our functional imaging data in more detail by quantifying the area and peak amplitudes of whisker representations at each time point. Prior to pMCAO, no differences between groups in area or peak amplitude of the ipsi-ischemic C2 representation were observed. Differences were found between the groups for baseline versus 24 hours for the area (F_2,18_ = 13.60, p = 0.00251, ANOVA) and peak amplitude (F_2,18_ = 31.45, p = 0.00001, ANOVA). Both the area (F_1,18_ = 15.823, p = 0.000882) and amplitude (F_1,18_ = 50.655, p = 0.000001) of the ipsi-ischemic C2 representation had disappeared in +3 h animals 24 hours post occlusion (Figure 4A; for more inferential statistics also see Table S1). In +0 h animals, the area and amplitude was actually larger than baseline at 24 hours post occlusion, though only the peak amplitude increase reached significance (F_1,18_ = 8.70, p = 0.00855, Figure 4A, Table S1).

The functional imaging analyses presented thus far assessed the first (the initial dip) of three phases composing the whisker functional representation [18]. The remaining two phases (overshoot and undershoot) were also assessed. Within subjects, both phases behaved similarly to the previously described initial dip (Figure S3A and Table S1).

#### Normal evoked neuronal activity was maintained if animals received whisker stimulation immediately after pMCAO, but not if stimulation was delayed for 3 hours

**Figure 5:**
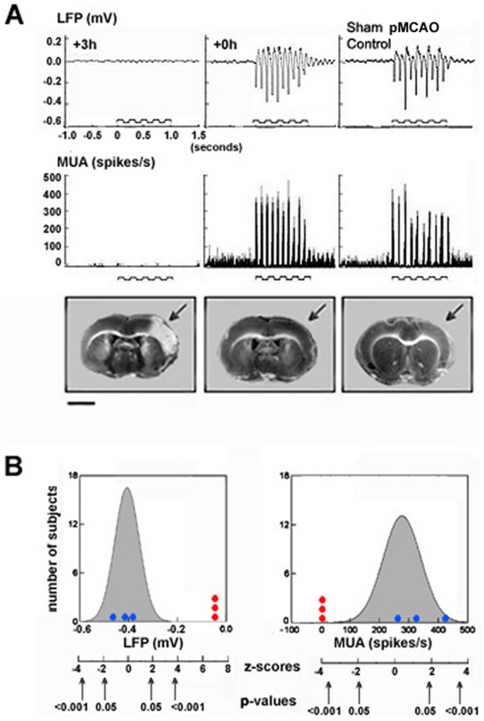
Normal evoked neuronal activity was maintained in all +0 h animals. (A) Representative +3 h, +0 h, and sham pMCAO control subjects' local field potential (LFP, first row) and multi-unit activity (MUA, second row) responses 24 hours following pMCAO. Stepping function indicates stimulus delivery. Representative coronal section taken from experimental and control animal's 2,3,5-Triphenyltetrazolium Chloride (TTC) assay for infarct. Area devoid of staining (arrows) within the +3 h subject's left cortex is indicative of ischemic infarct due to an occlusion of the left MCA. Scale bar indicates 5 mm. (B) LFP (left) and MUA (right) normal distributions based on sham pMCAO control data are plotted as shaded curves. Superimposed on these distributions are the individual values of +0 h subjects (blue circles) and +3 h subjects (red circles). Provided below each graph are the z-scores (as derived in relation to the normal distribution of sham pMCAO controls), and corresponding p-values.

We next corroborated our ISOI data with direct measurement of neuronal activity. Suprathreshold, multi-unit activity (MUA) and subthreshold, local field potential (LFP) were simultaneously recorded in additional rats. 24 hours after pMCAO, an electrode was inserted perpendicularly to the location of peak evoked activity over the ipsi-ischemic C2 whisker barrel (determined the previous day using ISOI) and recordings were obtained within layers II/III (∼300–400 µm, supragranular layer). Rats were divided into +0 h (n = 3, Figure 1J) and +3 h (n = 3, Figure 1K) groups. The same stimulation and data collection parameters that were used for imaging were used for these recordings. Congruent with imaging and TTC findings for +3 h animals (Figure 3 and 4), no suprathreshold and extremely weak subthreshold neuronal responses were evoked in +3 h rats, and infarct was present 24 hours post-pMCAO (Figure 5A). In contrast responses recorded from sham pMCAO control animals (n = 21, Figure 1L) were robust (sham pMCAO control animals underwent the same surgery and protocol as +0 h animals [Figure 1A], except their suture threads were never tightened to occlude MCA) (Figure 5A). Neuronal responses of +3 h rats were converted to z-scores relative to the distribution of sham pMCAO control animal values, which ranged from −4.10 to −4.32 for the suprathreshold responses and 7.0 to 7.54 for the subthreshold responses, all corresponding to p-values<0.0001 based on a normal probability distribution (Figure 5B, red circles). In contrast, consistent with our imaging and TTC findings (Figure 3 and 4), +0 h animals exhibited robust suprathreshold and subthreshold evoked responses and did not sustain infarct (Figure 5A). The +0 h animals' evoked responses fell within the distribution of sham pMCAO control group values: the z-scores ranged between −0.18 to 2.36 for the suprathreshold and −1.45 to 0.30 for subthreshold responses (Figure 5B, blue circles). These results are highly consistent with our ISOI results, and serve as additional confirmation that the cortices of +3 h rats lose function as a result of ischemic injury, while the cortices of +0 h rats are protected from ischemic injury and indistinguishable from controls.

#### Normal sensorimotor related behavior was maintained if animals received whisker stimulation immediately following pMCAO, but not if stimulation was delayed for 3 hours

**Figure 6:**
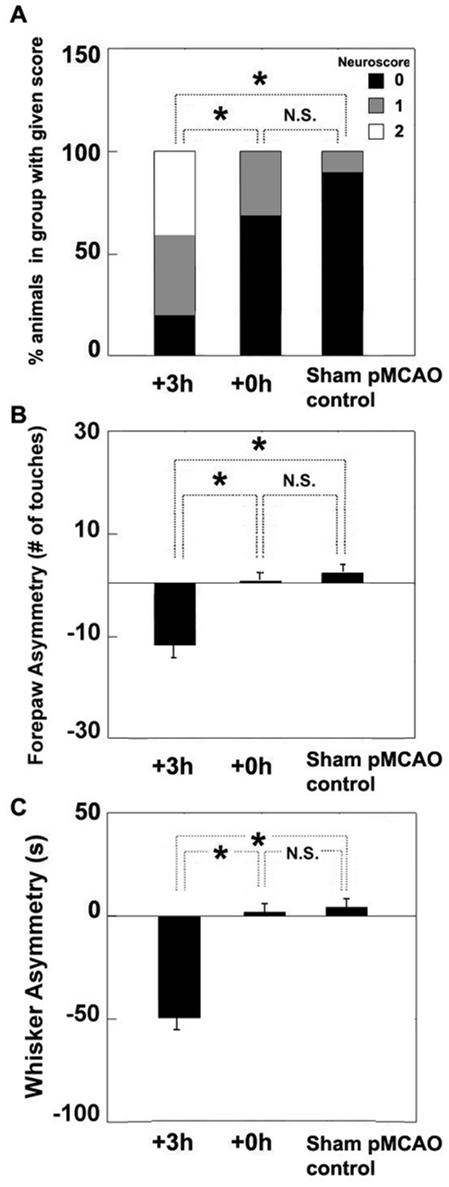
Normal sensorimotor-related behaviors were maintained in +0 h animals. All +3 h animals (n = 22) exhibited behavioral deficits 7 days after the onset of ischemia (time of pMCAO), while +0 h animals (n = 22) were indistinguishable from controls (animals which underwent surgical preparation with no MCA occlusion, n = 22). Asterisks indicate significant differences between bracketed groups and N.S. indicates no difference between bracketed groups. (A) Neurological scores according to the Bederson Scale. Each color portion of each bar represents the percentage of animals within each group with the corresponding neurological score (see color key). (B) Use of left (ipsi-ischemic) versus right (contra-ischemic) forelimbs expressed as an asymmetry score during forepaw guided exploration. Forepaw asymmetry was calculated as total number of explorations initiated with the right forepaw minus total number of explorations initiated with the left forepaw. Horizontal line indicates ‘0’, or no asymmetry. (All three spent equivalent amounts of time exploring with their paws). (C) Use of left side (ipsi-ischemic) versus right side (contra-ischemic) whisker sets expressed as whisker asymmetry during whisker guided exploration. Whisker asymmetry was calculated as total time spent scanning with the right whisker set minus total time spent scanning with the left whisker set. Both +0 h and controls used their left and right whisker sets equally. Horizontal line indicates ‘0’, or no asymmetry. All three groups explored with their whiskers for equivalent amounts of time.

Each animal in each experimental group (+3 h, +0 h, and sham pMCAO controls, n = 22 per group, Figure 1M–O) was assessed using all three of the following behavioral tests 7 days after pMCAO: the *Bederson Neurological Scale* [19], [20], forepaw-guided exploration [21], and whisker-guided exploration [22], [23], [24]. Animals that did not receive whisker stimulation until three hours post-pMCAO (+3 h) performed significantly worse than sham pMCAO controls on the Bederson Neurological Scale (Pearson chi-square test [2, *N = 44*] = 24, p = 0.00001; Figure 6A). Differences were also found between groups for forepaw-guided exploratory behavior (F_2,63_ = 40.09, p = 2.0×10^−11^, ANOVA; Figure 6B). +3 h subjects increased the usage of their unaffected (left) forepaw when compared with sham pMCAO controls (F_1,63_ = 69.14, p = 2.0×10^−11^; Figure 6B). Lastly, a group difference was also found in whisker-guided exploration (F_2,63_ = 42.20, p = 2.0×10^−11^, ANOVA). +3 h rats showed an increase in dependence on the unaffected whisker pad compared to sham pMCAO controls (F_1,27_ = 20.10, p = 0.000122; Figure 6C).

+0 h subjects consistently demonstrated unimpaired behaviour on all tasks. These animals scored significantly better on the Bederson Neurological Scale when compared to +3 h subjects (Pearson chi-square test [2, *N = 44*] = 16.2, p = 0.0003; Figure 6A), and +0 h animals also demonstrated significantly less asymmetry when compared to +3 h subjects in forepaw (F_1,63_ = 49.51, p<0.0001; p = 2.0×10^−9^; Figure 6B) and whisker guided behavior (F_1,63_ = 58.27, p = 2.0×10^−10^; Figure 6C). Most notably, there were no differences in neurological score, forepaw-guided, or whisker-guided exploratory activity between +0 h subjects and sham pMCAO controls (Figure 6). Taken together, these data demonstrate that sensorimotor and exploratory behavior remains intact in animals that receive whisker stimulation immediately following pMCAO, but not in animals that do not receive whisker stimulation until three hours post-pMCAO.

Finally, all behavioral subjects were sacrificed at seven days post-pMCAO for post-mortem examination using TTC. All +3 h rats sustained an infarct in the ischemic cortex, but, as expected, none of the +0 h subjects showed any sign of infarct. Thus, +3 h rats failed to maintain normal sensorimotor, forepaw-guided, and whisker-guided behavior one week following ischemic injury and sustained infarct. In contrast, +0 h animals demonstrated intact sensorimotor, forepaw-guided, and whisker-guided exploratory behaviors indistinguishable from controls, and sustained no infarct. These behavioral results further support our imaging, electrophysiological recording, and previous histological staining data, and all of these data taken together demonstrate that whisker stimulation is capable of completely protecting the cortex from ischemic injury.

#### Whisker stimulation protected functional representations and cortical tissue in all animals even when delivered one hour post-pMCAO and in most animals when delivered two hours post-pMCAO

The same protection evident when whisker stimulation was delivered immediately after pMCAO (in+0 h subjects) was also observed when whisker stimulation was not delivered until 1 hour after pMCAO (+1 h, n = 7; Figure 1C and Figure S2). +1 h animals maintained at least baseline level ipsi-ischemic C2 whisker representation and sustained no infarct. In +1 h animals, the area and the peak amplitude of C2 whisker representation were also larger than baseline levels at 24 hours post occlusion, though only the increase in area (rather than amplitude as seen in +0 h animals) reached significance (F_1,18_ = 7.92, p = 0.01147, (Figure S2, Table S1).

We further sought to address whether whisker stimulation at 2 hours post-pMCAO would still be protective. Another group of animals therefore underwent the same procedures as +0 h, +1 h, and +3 h animals except that whisker stimulation was not administered until 2 hours after pMCAO (+2 h group, n = 8, Figure 1D). Six of eight +2 h animals were functionally and structurally intact according to ISOI and TTC staining. Two animals, however, sustained infarct (though this infarct was smaller than that observed in +3 h animals) and had diminished (but present) whisker functional representation at 24 hours compared to baseline (Figure S2). Thus, when administered within an hour of pMCAO, whisker stimulation is completely and consistently protective, and when delivered 2 hours post-pMCAO, stimulation is still beneficial, but protection is no longer consistent.

### A return of blood flow to MCA (in the distal to proximal direction) via collateral vessels appears to be the underlying mechanism in the re-establishment of blood flow to the ischemic area

#### Distal MCA branches are necessary for the protection observed in animals that received whisker stimulation immediately following pMCAO

**Figure 7:**
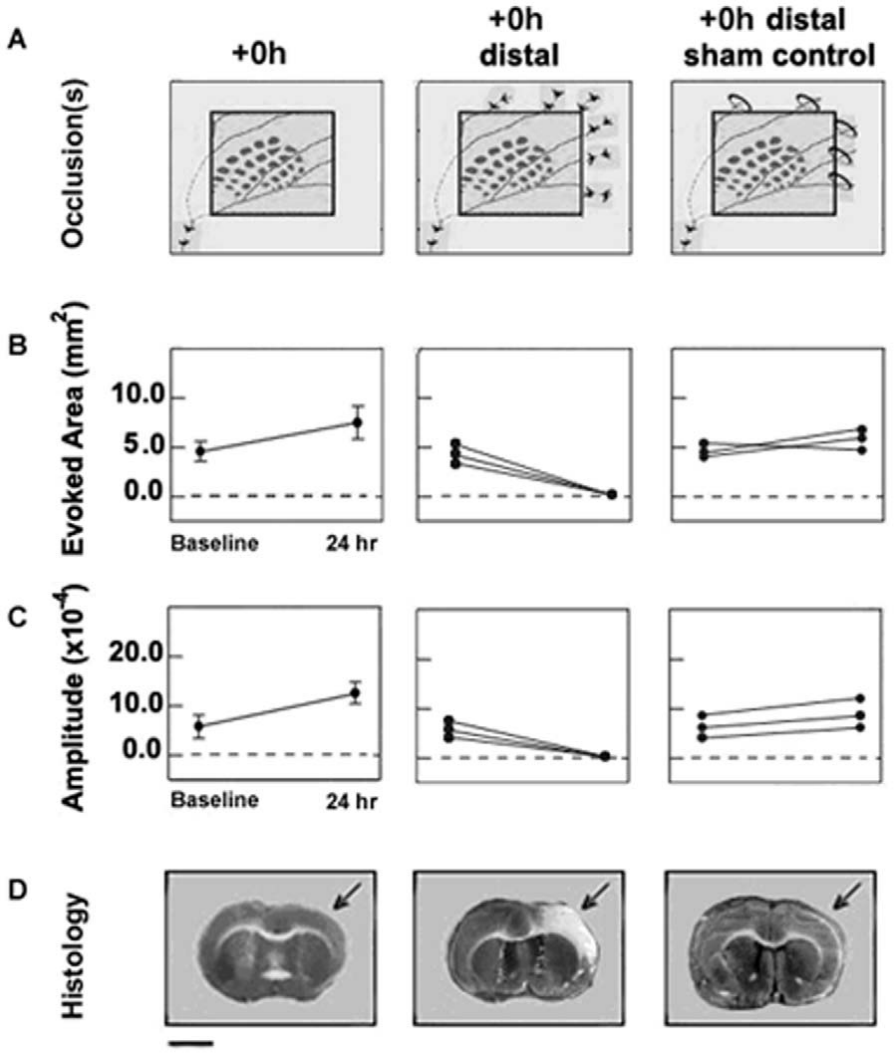
Distal occlusion experiments demonstrate that patent distal branches of MCA are necessary for protective plasticity. (A) Schematics of relative positions of barrel cortex, MCA, and occlusion(s) for +0 h (n = 7; left), +0 h distal (n = 3; middle), +0 h sham distal control (n = 3; right) groups. All three groups underwent identical protocols aside from the additional occlusions of distal branches of MCA performed in the +0 h distal group, and unsecured sutures over the same distal MCA branches in the +0 h sham distal control group. (B, C): Graphs Baseline and 24 hour means and standard errors for area (B) and amplitude (C) of ipsi-ischemic whisker C2 representation for +0 h group, and individual values for +0 h distal and +0 h sham distal control animals. (D): Below each set of graphs is a representative TTC stained coronal slice for each group. The area devoid of staining (arrows) within the +0 h distal subject's left cortex is indicative of ischemic infarct due to an occlusion of the left MCA. Scale bar indicates 5 mm.

Although the ISOI and electrophysiological data provide critical insights about functionality (for which blood supply is a pre-requisite), these data did not allow us to determine how blood flow was re-established following pMCAO. It is known that the distal ends of MCA branches can sometimes be connected to other arteries, forming collateral vessels (this occurs in both rodents and humans), and we posited that these could be a source of reperfusion into MCA cortical branches [25], [26]. To determine if blood could be flowing through these collaterals to re-establish blood flow to MCA territory, we imaged the ipsi-ischemic C2 representation in additional +0 h rats (+0 h Distal, n = 3) that underwent not only our standard pMCAO at the base of MCA but also underwent permanent occlusions at the distal ends of all main MCA cortical branches in a procedure similar to that described by Wei et al., 1995 [27] (Figure 1H and 7A). We reasoned that if blood reperfusion were established via collateral flow, this procedure would prevent such re-establishment and therefore prevent the protection previously observed in +0 h animals. Indeed, the ipsi-ischemic C2 representation disappeared and substantial infarct was observed in all +0 h distal subjects despite the fact that whisker stimulation was delivered immediately following occlusion (Figure 7, middle column). These results were in stark contrast to the protection of functionality and tissue health observed in +0 h animals that did not receive distal occlusions in addition to pMCAO (Figure 7, left column). Further, to control for possible damage caused by additional surgical procedures inherent in the +0 h distal animal experimental design, we assessed function in +0 h sham distal control animals. This group underwent the same surgical protocol (including permanent occlusion of MCA's base) as +0 h distal animals except that the distal occlusion suture knots were never tightened to occlude the distal ends of MCA branches (+0 h sham distal control animals; n = 3; Figure 1I and 7A). Despite having the same additional surgical intrusion as +0 h distal animals, these controls maintained normal whisker functional representation and tissue health according to ISOI and TTC staining (Figure 7, right column). Taken together, these data suggest that the distal MCA branches are necessary for stimulation-induced protection of the cortex from ischemic injury.

#### Blood flow in MCA cortical branches was re-established by 24 hours post occlusion if animals received whisker stimulation immediately following pMCAO, but not if stimulation was delayed for 3 hours

**Figure 8:**
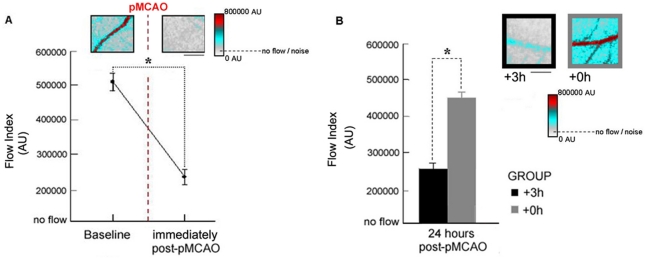
LSI experiments demonstrate that blood flow in MCA is re-established on the following day if whisker stimulation is administered immediately post-pMCAO. Insets, Representative linearly color scaled LSI images taken at (A) pre-pMCAO baseline and immediately post-pMCAO, or (B) 24 hours post-pMCAO for an animal from each of +0 h and +3 h groups. Scale bar indicates 0.25 mm. Graphs, the x-axis crosses the y at the mean noise level, or, ‘no flow’ level as determined by averaging values recorded from a group of euthanized animals (n = 6) at approximately 5 minutes after cessation of heart beat (MCA was thus no longer flowing in these animals hence the designation ‘no flow’ for equivalent values). To establish flow values in the absence of flow (noise) we collected data from a group of euthanized animals 5 minutes after the cessation of heart beat. We analyzed an MCA ROI as we had in live subjects and determined an average noise or ‘no-flow’ value (106,595±12,481SE). (A): Inset, note the large reduction in MCA flow. Graph, means and standard errors for MCA flow at pre-pMCAO baseline and immediately post-pMCAO. * indicates a significant difference between baseline and immediately post-pMCAO flow values (B): Inset, note the minimal blood flow apparent in the +3 h subject and the substantial blood flow in the +0 h subject despite the fact that the same double suture and complete transection of MCA's base was performed in both animals 24 hours prior. Graph, means and standard errors for MCA in +3 h and +0 h subjects 24 hours post-pMCAO. Note that the minimal flow values in +3 h group animals at 24 hours post-pMCAO are similar to levels seen immediately post-pMCAO in animals in panel A and that the flow values in +0 h animals are similar to baseline flow levels in panel A. * indicates a significant difference between +0 h and +3 h flow values at 24 hours post-pMCAO (*p = 0.000005). Note that pMCAO was performed by tightening two suture knots around the base of MCA and then completely transecting the artery between these two knots.

To further investigate the role of MCA in reperfusion, we directly imaged changes in blood flow in MCA's cortical branches using laser speckle imaging (LSI). First we analyzed pre-pMCAO baseline data and immediately post-pMCAO data for a group of animals (n = 13, Figure 1P). This allowed us to determine average pre-occlusion flow values (an equivalent to measuring sham pMCAO controls) and also to assess the impact of our occlusion on flow values in MCA. Robust blood flow was observed in the cortical branches of MCA prior to pMCAO; these flow levels were reduced significantly from baseline (paired t-test, t(12) = 7.75, p = 0.000005; Figure 8A) and approached noise levels immediately following the occlusion.

Second, we analyzed and compared flow value data for +0 h (n = 6, Figure 1Q) and +3 h (n = 6, Figure 1R) animals at 24 hours post-pMCAO to assess the difference in blood flow in protected +0 h animals versus non-protected +3 h animals. By 24 hours post-pMCAO, +0 h subjects demonstrated significantly higher post-pMCAO flow when compared to +3 h subjects (unpaired t-test, t(10) = 8.79, p = 0.000005; Figure 8B), and these +3 h animals also, as expected, sustained cortical infarct. Note that the flow values of +0 h animals at 24 hours (Figure 8B, gray bar) are similar to flow values recorded pre-occlusion (Figure 8A). Also note that the flow values of +3 h animals at 24 hours (Figure 8B, black bar) resemble the reduced flow observed immediately after pMCAO was administered (Figure 8A). These data demonstrate that when whisker stimulation is administered immediately after pMCAO, normal blood flow levels are re-established within the ischemic cortex by the following day.

Directionality of blood flow cannot be determined with LSI, but because pMCAO included a *complete transection* of the base of MCA (between the two tightened suture knots), blood flow observed in the cortical branches of MCA (distal to the occlusion) at 24 hours post occlusion, must be flowing (in the reverse direction) from another arterial source via collateral vessels. In conjunction with the distal experiments discussed above, these data suggest that a mechanism underlying the observed protection from ischemic injury is likely a redirection of blood from an alternate source, perhaps the Anterior Cerebral Artery, via collaterals to MCA, allowing re-establishment of blood flow to the ischemic cortex. Spontaneous blood flow reversal has been demonstrated recently in small surface arteries of the rodent somatosensory cortex following MCAO [26], [28]; such reversals are highly supportive of cortical integrity during post-ischemic reperfusion of areas surrounding infarcted regions (penumbra) [29]. We now report a large scale stimulus dependent blood flow reversal in the largest branches of MCA, demonstrating that when whisker stimulation is delivered within an hour of ischemic onset, the cortex is capable of plasticity involving a major cortical artery.

### Whisker stimulation protects additional (non-stimulated) ischemic whisker representations and effects are limited to the ischemic hemisphere

#### Whisker functional representations of non-stimulated whiskers were also preserved in the ischemic cortex and no changes were observed in the contra-ischemic cortex

To further elucidate the ability of whisker stimulation to provide protection, we addressed the following two questions: 1) The stimulated whisker's representation was preserved in our +0 h and +1 h rats. Were the representations of ipsi-ischemic but non-stimulated whiskers preserved? 2) Were effects limited to the ischemic hemisphere?

To address the first question we assessed the functional representation of an additional whisker (whisker B1) in the ischemic cortex (at 24 hours post-pMCAO) that had not received stimulation post-pMCAO (n = 5 per group). The same overall ISOI results were obtained for whisker B1 as described for C2 (Figure S3B), suggesting that stimulation of a single whisker is sufficient to protect not only that whisker's functional representation, but also those of neighboring whiskers.

To address the second question and determine whether changes in the C2 representation reflected a bilateral change in cortical function, we measured the contra-ischemic C2 representation before and 24 hours after pMCAO and found that it remained unchanged in all animals (Figure S3C), suggesting that the observed differences between the three experimental groups were constrained to the ischemic hemisphere, at least by 24 hours.

## Discussion

Results from functional imaging, neuronal recording, histological assay, behavioral assessment, and blood flow measurements collectively suggest that protective plasticity induced by mild, intermittent sensory stimulation can completely protect the cortex from impending ischemic injury. The distal experiments and blood flow imaging further suggest that when whisker stimulation is administered within a critical time window, normal blood flow levels are re-established within the ischemic cortex via blood flow back into the branches of MCA in the reverse direction, flowing from an alternate arterial source via collateral vessels. Because stimulation at 3 hours after pMCAO not only failed to protect the cortex, but induced an even larger infarct than when stimulation was never delivered, our results further indicate that there is a limited time window during which such stimulation induced plasticity is beneficial. We also performed anesthesia matched controls for each experimental group in order to confirm that varying lengths of time under anesthesia independent of whisker stimulation did not affect outcome. Additionally, we confirmed using ISOI that protection was granted not only to the stimulated whisker but also to other ipsi-ischemic whiskers. Finally, we determined that stimulation does not induce any detectable changes in whisker representation (according to ISOI) in the contra-ischemic hemisphere, at least not by 24 hours post pMCAO.

How might the intermittent stimulation of a single whisker account for plasticity within such a large volume of cortex? We have recently reported that single whisker stimulation activates a very large symmetric subthreshold response area of ∼38.5 mm^2^ and a symmetric volume of ∼63 mm^3^ by spreading evoked activity via an underlying network of long-range horizontal connections [30]. The volume of 63 mm^3^ of activation exceeds the average infarct volume obtained in no-stimulus controls (28.4 mm^3^), and therefore, if this activity is directly responsible for the protection, its spread could account for the ability of single whisker stimulation to protect such a large cortical volume. Interestingly, cortical activation is also harmful if delayed for too long after ischemic onset. An activation volume of 63 mm^3^ seen in response to single whisker stimulation is very similar to the average infarct volume obtained in the +3 h group (61.4 mm^3^). Thus the volume of subthreshold cortical activation resulting from whisker stimulation ‘maps’ onto the volume of infarct seen in +3 h animals. The large volume of cortical activation by single whisker stimulation could therefor account for both the size of protection in the +0 h group, +1 h group, +2 h protected individuals, and for the size of the infarct in +3 h group.

Our findings suggest a surprising and critical role for cortical activation via non-invasive sensory stimulation in determining outcome following an ischemic event. Both the rodent and human brain have a remarkable capacity for plasticity which can facilitate rehabilitation after various forms of brain injury, including that caused in models of Parkinson's disease [31], traumatic brain injury [32], and stroke [33], [34]. In recent literature, plasticity has even been shown to reduce the degree of impending ischemic damage when induced shortly after ischemic onset using invasive and semi-invasive stimulation of the peripheral nerves [35], brain [36], and spinal cord [37]. Our work indicates that plasticity can not only induce a reduction in damage, but can in fact provide complete protection from ischemic injury. Furthermore the present study suggests that the cortex is even more sensitive to the induction of such plasticity than previously suspected - it can be initiated via a subtle and non-invasive means of cortical activation: mild sensory stimulation.

Our study suggests a critical time window following ischemic onset during which whisker stimulation can result in both protection of the cortex and robust reperfusion of the ischemic area. Additionally there is evidence in the human and animal model literature suggesting a similar time window during which reperfusion can reduce damage following ischemic onset. A recent study found that reperfusion after a transient (∼7 minute) global ischemia restored cortical function and reversed early damage [38]. Also, recent *in vivo* analysis demonstrates that a reperfusion of blood enables some reversal of dendritic damage even 1 hour after a reversible MCAO [28]. Perhaps the protection induced by whisker stimulation in our study results from a similar restoration of cortical function and reversal of damage via reperfusion even at one and two hours post-ischemic onset. The fact that no evidence of ischemic injury or stroke was observed in any animal that received whisker stimulation within an hour of pMCAO (all +0 h and +1 h animals, n = 45), but that a small subset of +2 h animals (2 of 8) that did not receive whisker stimulation until 2 hours after pMCAO sustained infarct and had decreased whisker functional representation, suggests that whisker stimulation is most effective up until some point between 1 and 2 hours post ischemic onset. This critical time window for successful intervention is consistent with the accumulated human ischemic stroke data which suggest that thrombolysis treatment, which causes reperfusion, is most effective if given within 1.5 hours of symptom onset [39].

The distal experiments in this study demonstrate that the distal branches of the permanently occluded MCA are necessary for sensory induced protection. Additionally, our blood flow imaging results suggest that reperfusion occurs via MCA branches; given the transection of MCA's base, the only explanation for the flow return evident in +0 h animals' MCA branches is collateral connections allowing flow from an alternate arterial source. Our work therefore demonstrates that collateral vessel connections play a critical role in this reperfusion, which is obviously a necessary component of protection from ischemia. Such vascular redundancy is also known to occur in the human brain [20] suggesting that the induction of similar protection might be feasible in a clinical setting.

The age of the animals in our study (3–4 months) is a potential limitation given that rats of this age are equivalent to young adult humans – a population that rarely suffers from ischemic stroke. While stroke can occur at any age, 72% of stroke victims are over the age of 65 [20]. Preliminary findings from our laboratory suggest, however, that the same single whisker induced neurovascular plasticity is also fully protective in aged rats (20–21 months old), indicating that our findings may also be relevant for more vulnerable aged populations.

In conclusion, this study indicates that a relatively modest intervention, intermittent stimulation of a single whisker, if timed appropriately, can have the dramatic effect of preventing the cortical consequences of ischemia. These data will now allow us to explore the cellular and molecular mechanisms of the time-sensitive events that underlie this protection. An understanding of the mechanism by which stimulation facilitates sufficient reperfusion and protection from stroke could have significant clinical potential. For example, such understanding could lead to the development of drugs that imitate the protective effect of sensory stimulation. Alternatively, though humans lack whiskers, regions of the body that have a large representation in the human somatosensory cortex such as digits and lips could be potential targets for tactile stimulus during an ischemic episode. These results also raise the possibility that sensory stimulation could be applied to suspected stroke victims during, or even before their transfer to emergency facilities - an approach that is not only simple and non-invasive, but also compliments recent calls for early intervention for stroke victims [40].

## Materials and Methods

### Ethics Statement

All procedures were in compliance with NIH guidelines and approved by UC Irvine Animal Care and Use Committee (protocol #: 1997-1608, assurance ID#: A3416.01).

### Subjects and surgical preparation

Experimental subjects, 295–400 g male Sprague Dawley rats (Charles River Laboratories, Wilmington, MA, USA), were individually housed in standard cages. At the beginning of each experiment, subjects were injected intraperitoneally with a Nembutal bolus (55 mg/kg b.w.). Supplemental injections of Nembutal (27.5 mg/kg b.w.) were given as necessary. After resection of soft tissue, a ∼6.5×8 mm ‘imaging’ area of the skull over each (left and right) primary somatosensory cortex (rostromedial corner positioned approximately 1 mm caudal and 2 mm lateral from bregma) was thinned to ∼150 µm using a dental drill. 5% dextrose (3 mL) and atropine (0.05 mg/kg, b.w.) were administered at the beginning of the experiment and every six hours after until the animal was returned to its home cage (the first day of each experiment typically lasted 8 to 10 hours and the second day typically lasted 2 to 4 hours). Body temperature was measured via a rectal probe, and maintained at 37° Celsius by a self-regulating thermal blanket. After the completion of the experiment, all animals were returned to their home cage and allowed to recover overnight prior to all +24 hour experimentation.

### Overview

Functional Imaging, neuronal recording, behavior, blood flow imaging, and control groups' experimental timelines are summarized in Figure 1. All experimental subjects underwent pMCAO. Following pMCAO, all experimental subjects were randomly assigned to one of the following experimental groups: +0 h, +1 h, +2 h, or +3 h. Groups are so named for the latency (in hours) between pMCAO and the initiation of post-occlusion whisker stimulation. Post-occlusion whisker stimulation consisted of 1 s of 5 Hz deflections of a single whisker (whisker C2). This stimulation was intermittently (with random intervals averaging 21 seconds) delivered 256 times, totaling 4.27 minutes of stimulation, over the course of 2 hours. After whisker stimulation, animals were returned to their home cage and allowed to recover. Rats in functional imaging (Figure 1A–I), electrophysiological recording (Figure 1J–L), or blood flow imaging (Figure 1P–R) groups were assessed at 24 hours post-pMCAO. Behavioral group rats (Figure 1M–O) were tested at 7 days post-pMCAO. At the conclusion of each experiment, all rats were sacrificed and TTC staining was used to assess cortical tissue health. Various additional experimental controls were also performed. (See below for detailed methodology and experimental design).

### Permanent Middle Cerebral Artery Occlusion (pMCAO)

Ischemic conditions were achieved via surgical occlusion of the stem of the left proximal middle cerebral artery [20], [41], [42]. The skull and dura were carefully removed from a 2×2 mm ‘surgical window’ just anterior and lateral to the imaging window (over MCA's stem; or the M1 segment just distal to MCA's lenticulostriate branch) and a half-curve reverse cutting suture needle and thread (4–0 silk) was passed through the pial layer of the meninges, below MCA and above the cortical surface. In the first 9 of 36 ISOI experiments conducted, a single thread was tied around the MCA with a surgical knot. Then, to further ensure that our pMCAO had completely and permanently obstructed blood flow, we performed a double ligature technique in the remaining ISOI experiments and all subsequent experiments (electrophysiology, distal ligatures, LSI, behavioral experiments): two threads ∼1 mm apart were tied and tightened around MCA and the vessel was then transected (completely severed) between the two knots. Because results were found to be the same using either procedure, the single and double ligature ISOI experiments were pooled together and treated as one data set. Care was taken to avoid damaging the artery, and experiments were terminated if there was any sign of bleeding from MCA. Three animals were disqualified from this study due to obvious arterial abnormalities or malformations [43], [44]. (Also, in a subset of animals, Arterial O_2_ saturation, respiration, and heart rate were measured using the MouseOx pulse oximetry unit (Starr Life Sciences, Allison Park, PA) to ensure that observed changes following pMCAO were not due to variability in vital parameters due to changes in anesthesia level).

### Histology

Rats were euthanized with 2.0 ml of Euthasol at the conclusion of each experiment. Their brains were carefully removed and sectioned into 2 mm slices along the coronal plane. The brain slices were then incubated in 2% 2,3,5-triphenyltetrazolium chloride (TTC; Sigma, St. Louis, MO, USA) at 37°C for twenty minutes in the dark [19]. TTC is enzymatically reduced, producing formazan (a bright red byproduct), by dehydrogenases in active mitochondria. Red stain intensity correlates with the number and functional activity of mitochondria, unstained (white) areas are indicative of infarct [11], [45]. The TTC-stained sections were photographed with a digital camera, and the total infarct volume was determined by multiplying the infarct area of each slice by the thickness of that slice. An observer blind to experimental condition performed this volume calculation. Within each group, subjects' TTC images were digitally superimposed and traced onto a group coronal brain map. These traces collectively formed an experimental group composite from which common location and overlap (between individuals within the same group) was ascertained. A small lesion (<1 mm in diameter) was occasionally apparent at the immediate site of MCA occlusion. This occurred infrequently and equivalently in all experimental groups (1–2 subjects per group). The small amount of damage occasionally produced at the surgical site could be readily distinguished from the large ischemic infarct and was excluded from infarct analysis [41].

### Intrinsic Signal Optical Imaging (ISOI) and Analysis

A detailed description of ISOI [14], [15], [16] data acquisition and analysis can be found elsewhere [18], [46]. Briefly, a charge coupled device (CCD) camera (either a 16-bit Cascade 512F or a 12-bit Quantix 0206, Photometrics, Tucson, AZ, USA) equipped with an inverted 50 mm AF Nikon lens (1∶1∶8, Meliville, NY, USA) combined with an extender (model PK-13, Nikon, Meliville, NY, USA) was used for imaging and controlled by V++ Precision Digital Imaging System software (Digital Optics, Auckland, NZ). During each 15-s trial, 1.5 s of prestimulus data followed by 13.5 s of poststimulus data were collected, with a 6±5 sec random inter-trial interval (total time between one trial onset and the next was therefore 21 seconds on average). Stimulus consisted of a single whisker being deflected by 9° in the rostral-caudal direction at a rate of 5 Hz for a total stimulus duration of 1 second. (Note that this stimulation protocol is used whenever whisker stimulation is administered at any point for any experiment in this manuscript). The cortex was illuminated with a red light emitting diode (635 nm maximum wavelength with full width at half height of 15 nm). Data were collected in blocks of 64 stimulation trials, and a sampled time point (for example pre-pMCAO baseline) was considered complete upon summation of 128 stimulation trials. Ratio images were created from calculating fractional change (FC) values by dividing each 500 ms frame of poststimulus signal activity by the 500 ms frame of prestimulus intrinsic signal activity collected immediately before stimulus onset. The ratio image containing the maximum areal extent for each of the three intrinsic signal phases (Initial Dip, Overshoot, and Undershoot) was Gaussian filtered (half width = 5) and the areal extent quantified at a threshold level of 2.5×10^−4^ away from zero. Peak amplitude was quantified in fractional change units from the pixel with the peak activity within the maximum areal extent for each of the three intrinsic signal phases. To explicitly verify the absence of any whisker functional representation, the very low threshold 0.5×10^−4^ (close to absolute zero) was used to qualitatively examine all images for any sign of evoked activity (Figure S1).

Our main intention was to determine the effect of whisker stimulation on outcome following pMCAO. We also, however, performed a number of additional experiments to establish the effect of a delay between pMCAO and this whisker stimulation, so that, if there was indeed a protective effect, we could establish a time window for its induction. +0 h, +1 h, +2 h, and +3 h ISOI groups are so named for the number of hours between pMCAO and whisker stimulation (+0 h receives whisker stimulation immediately following pMCAO and +3 h has a 3 hour delay and so on). Additional control experiments were also performed. All ISOI groups in addition to control groups are outlined below.

### ISOI groups

**+0 h group** (Figure 1A, n = 7) animals underwent approximately 2 hours of baseline ISOI and subsequent pMCAO immediately followed by 2 hours of C2 whisker stimulation. These animals were then allowed to recover in their home cages. 24 hours later they were re-anesthetized, imaged using ISOI and sacrificed so that TTC staining could be used to assess cortical tissue health.

**+1 h group** (Figure 1C, n = 7) animals underwent the exact same protocol as the +0 h animals except that there was a **1 hour delay** between pMCAO and whisker stimulation.

**+2 h group** (Figure 1D, n = 8) animals underwent the exact same protocol as the +0 h animals except that there was a **2 hour delay** between pMCAO and whisker stimulation.

**+3 h group** (Figure 1E, n = 7) animals underwent the exact same protocol as the +0 h animals except that there was a **3 hour delay** between pMCAO and whisker stimulation.

**+0 h no stimulation control group** (Figure 1B, n = 3) animals and **+3 h no stimulation control group** (Figure 1F, n = 5) animals were matched to the +0 h and +3 h animals, respectively, for time spent under anesthesia, but never received whisker stimulation (though both groups did receive pMCAO). The purpose of this group was to determine whether length of time under anesthesia was a confounding variable.

**24 hr no stimulation control group** (Figure 1G, n = 2) animals were anesthetized and pMCAO was performed. To prevent self-stimulation of whiskers when the animals recovered from anesthesia in their home cages, these animals were kept under anesthesia for the entire 24 hours following pMCAO and were then sacrificed so that TTC staining could be used to assess cortical tissue health.

The purpose of this group was for comparison to +3 h animals who received whisker stimulation at 3 hours post-pMCAO in order to assess whether this late whisker stimulation was actually more harmful than no whisker stimulation at all.

### Electrophysiology and Analysis

Prior to pMCAO and electrophysiological recording, ISOI was performed to identify the location of peak optical activity evoked by whisker C2 stimulation in order to guide proper placement of electrodes for subsequent neuronal recording [47], [48]. Electrophysiology +0 h and +3 h animals underwent the same first day experimental protocol as ISOI +0 h and +3 h animals. The following day they were re-anesthetized, the thinned skull region and underlying dura removed, the cisterna magnum drained of cerebrospinal fluids to minimize edema, and the exposed cortex covered with silicon oil. A Tungsten microelectrode (∼1.5 mΩ) was inserted into the exposed cortex such that the electrode entered perpendicular to the location of peak optical activity determined the day before. We used data acquisition hardware and software from Alpha-Omega (Nazareth, Israel). Simultaneous recordings of both suprathreshold (multi-unit activity - MUA) and subthreshold (local field potential - LFP) evoked neuronal activity were obtained from a depth of ∼300–400 µm (supragranular layer) as measured from the cortical surface while the electrode penetrated the cortex using a micropositioner with 1 µm resolution steps. Recorded signals were amplified and bandpass (1–3000 Hz) filtered to allow simultaneous capture of MUA and LFP from the same electrode, and then digitized at 24 KHz rate. Real time traces and multi-unit peri-stimulus time histograms (PSTH) were generated to monitor quality and consistency of recordings. One complete recording session consisted of 64 whisker stimulation trials using the same stimulation parameters as those used during ISOI. Each trial contained 1.5 s of baseline activity followed by 13.5 s of post-stimulus onset activity. Spike2 software (Cambridge Electronic Design, Cambridge, UK) was used for the off-line extraction of MUA and LFP by re-filtering the collected data in either the 1–300 Hz range (LFP) or 300–3000 Hz range (MUA), for the subsequent analysis. A single average LFP waveform or MUA PSTH in 1 msec bins was generated from the 64 stimulation trials. LFP magnitude was then calculated as the mean of all 5 peak ‘on’ responses minus the mean obtained from 1-sec duration of pre-stimulus data. Similarly, MUA magnitude was calculated as the mean firing rate (spikes/s) of all 5 peak ‘on’ responses minus the mean firing rate obtained from 1-sec duration of pre-stimulus data. Electrophysiological groups and controls are outlined below.

### Electrophysiology groups

**+0 h group** (Figure 1J, n = 3) animals underwent approximately 2 hours of baseline ISOI (to locate peak activity in response to whisker stimulation). This was followed by pMCAO immediately followed by 2 hours of C2 whisker stimulation. These animals were then allowed to **recover** in their home cages. The following day they were re-anesthetized **and** electrophysiological data were collected. They were then sacrificed and TTC staining was performed to assess cortical tissue health.

**+3 h group** (Figure 1K, n = 3) animals underwent the exact same protocol as the Electrophysiology +0 h animals except that there was a **3 hour delay** between pMCAO and whisker stimulation.

**Electrophysiological sham pMCAO control group** (Figure 1L, n = 21) animals underwent the exact same protocol as the Electrophysiology +0 h animals except that the occlusion knots were never pulled closed over MCA. The purpose of this group was to control the effect of surgery and protocol aside from pMCAO might have on evoked neuronal activity.

### Behavior and Analysis

The three behavioral tests were conducted seven days post-pMCAO. All animals were given five minutes to habituate to the testing room, and then behavior performance was recorded using a digital camera suspended above the testing apparatus, which was dimly lit with red light illumination. Multiple individuals, blind to the experimental group identity of the rats, performed the behavioral testing and analysis.

### Behavioral Tests

#### Bederson Neurological Scale

The following assessment scale, first introduced as the Bederson scale [19], was used to assess the general mobility of all subjects. Animals are first held by the base of the tail and suspended 2.5 meters above the floor for 10 seconds in order to determine whether the rat withdraws his affected limb at the shoulder, and/or elbow joint, and/or wrist. This withdrawing behavior is known as limb flexion, and is a recognized sign of ischemic injury. Next, the subject is placed directly into a large cylindrical chamber and allowed to roam freely for five minutes. Scorers look for signs of spontaneous circling behavior, difficulty with gait, and difficulty remaining upright. Results of the observation period (suspension and spontaneous movement) are scored on a 0–4 scale as follows:

0: Normal movement, 1: Failure to extend forepaw contralateral to infarct; this level of impairment is typically seen in animals with a mild focal lesion, 2: Circling locomotive behavior; suggestive of a moderate focal lesion, 3: Falling to the side of infarct: occurs in animals with a severe, but focal, lesion, 4: Lack of spontaneous movement or stupor; associated with very severe lesions (from Wang-Fischer, 2009 [20]).

#### Forepaw-guided Exploration

Each rat was placed in the center of a testing cylinder (20 cm in diameter and 45 cm in height) for five minutes. Several behaviors were scored during the five minute session: initiation of a wall touch following rearing using the left forepaw, right forepaw, or both paws together, and the lateral (exploratory) movements along the wall surface following a rear with either the left forepaw, right forepaw, or both paws together. Forelimb use was analyzed by viewing rat exploratory activity. Wall and exploration touches were calculated, and forepaw use was expressed as a forepaw asymmetry score (right paw touches minus left paw touches), with a negative score signifying a subject's preference to explore with the left forepaw. In normal subjects, there is a roughly even distribution of usage between left and right paws, while unilateral damage to the somatosensory cortex will result in a greater dependence upon the unaffected limb [21].

#### Whisker-guided Exploration

Each subject was placed in a 25-cm-wide rectangular track (120×80 cm, outer diameter) and was allowed 10 s to acclimate before the start of the 5 minute testing session. Whisker scanning was defined as the time spent by the subject touching the walls of the rectangular track with one set of whiskers while locomoting [23]. Care was given to exclude incidents such as rearing and grooming as well as exploration which involved scanning with both sets of whiskers simultaneously, as in the case when a rat is facing perpendicular to a wall surface. Scanning was measured in seconds spent using either the left or right whisker pad by a timer watching the recorded testing session. Each subject was then assigned a thigmotactic scanning score (right score minus left score), with a negative score signifying a subject's preference to scan with the left set of whiskers. While healthy animals occasionally exhibit a whisker set preference, averages across groups of animals do not suggest a disproportionate preference for one whisker set over the other. Animals with unilateral damage to the somatosensory cortex, however, show a preference only for the unaffected whisker set [24]. Behavioral groups and their controls are outlined below.

### Behavioral groups

**+0 h group** (Figure 1M, n = 22) animals underwent pMCAO which was immediately followed by 2 hours of C2 whisker stimulation. Animals were allowed to **recover** in their home cages. At 7 days post-pMCAO animals were assessed using the described behavioral tests, sacrificed and TTC staining performed to assess cortical tissue health.

**+3 h group** (Figure 1N, n = 22) animals underwent the exact same protocol as the +0 h behavior animals except that there was a **3 hour delay** between pMCAO and whisker stimulation.

**Behavior sham pMCAO control group** (Figure 1O, n = 22) animals underwent the exact same protocol as the +0 h behavior animals except that the occlusion knots were never pulled closed over MCA. The purpose of this group was to control for any effect that surgury or protocol aside from pMCAO might have on behavioral outcome 7 days after surgery.

### Distal Occlusion ISOI Experiments and Analysis

To test our hypothesis that collateral flow through distal MCA branches from an alternate arterial source was the mechanism allowing reperfusion of the ischemic cortex in saved animals, we performed a series of experiments in which (in addition to pMCAO of the proximal MCA stem) distal branches of MCA were also occluded (+0 h distal group). Aside from the additional distal occlusions, +0 h distal group animals underwent the same protocol as the ISOI +0 h animals. Additional control experiments were also performed. All groups are outlined below.

### Distal Occlusion ISOI Groups

**+0 h distal group** (Figure 1H, n = 3) animals underwent the same protocol as other +0 h ISOI animals except that the distal ends of MCA branches were also exposed and permanently occluded.

**+0 h sham distal control group** (Figure 1I, n = 3) animals underwent the same protocol as the +0 h Distal animals (including pMCAO at MCA's base) except that the knots around the distal ends of MCA branches were never tightened to occlude the distal vessels. The purpose of this group was to control for possible surgical trauma introduced by the protocol for the +0 h distal animals.

### Laser Speckle Imaging (LSI) and Analysis

During Laser Speckle Imaging (LSI) [49], [50] data collection, a 632.8 nm 15mW HeNe laser (Melles Griot, Albuquerque, NM, USA) was used as the illumination source. The laser beam was first expanded with a 2X lens in order to illuminate the thinned skull region of ∼25 mm^2^ in a more uniform manner. Care was taken to maintain the same level of illumination intensity over the imaged area of interest within each experiment. The speckle pattern from the 5.12 mm×5.12 mm imaged region was captured as 512×512 pixel images by a 16-bit CCD camera (Cascade 512F, Photometrics, Tucson, AZ, USA) equipped with a Navitar zoom lens plus extenders (1^st^ Vision, Andover, MA, USA). The frame exposure time for each image was 1 ms, and 10 consecutive images spaced 1.5 seconds apart were collected per time point. Immediately prior to collection of each set of 10 images, the laser beam was temporarily blocked with a shutter and a green LED was used as an illumination source to capture an image of the cortical surface for later visualization of the cortical vasculature and localization of regions of interest (MCA branches) for analysis. Collected images were processed as previously described [51]. For each time point, the 10 raw speckle images were converted to speckle contrast images by calculating the coefficient of variation (SD/mean) for the center pixel within 5×5 pixel sliding windows in each image. The resulting 10 speckle contrast images were then converted to speckle index images by calculating their inverse squares multiplied by the exposure time in seconds, so that larger index values corresponded to faster blood flow. Lastly, the 10 speckle index images of each sampled time point were averaged to improve signal-to-noise ratio.

To quantify blood flow within specific MCA branches downstream from the occlusion for each sampled time point, we calculated the mean value within a region of interest (ROI) as defined according to several criteria. First, the MCA ROI was required to be within the largest region that received laser illumination sufficient for analysis (>7% of camera maximum saturation level) at all sampled time points. Additionally, the MCA ROI could not include portions of the vessel that overlapped with other large vessels (identified by examination of reference images captured with green LED illumination). (This restriction was designed to prevent flow values from the overlapping vessel from confounding our measurement of MCA flow). Eligible regions were also required to be located distal to the initial branching of two common anteriorly projecting MCA branches. MCA ROI sizes ranged from approximately 200–1000 pixels (or 0.02–0.1 mm^2^) per animal depending on the amount of MCA visible withn the above stated contraints. All LSI subjects underwent pMCAO and whisker stimulation was administered using the same protocol as that used in ISOI experiments.

To determine noise level values we also imaged a subset of euthanized animals at 5 minutes after the cessation of heart beat and averaged the LSI flow values we collected from the non-flowing MCA (termed noise or ‘no flow’ values). Analysis of an MCA ROI was performed for these subjects in the same manner as described for live subjects. All LSI groups are outlined below.

### LSI groups

**LSI baseline and post-pMCAO group** (Figure 1P, n = 13) animals underwent the exact same pre-pMCAO protocol as the +0 h LSI and +3 h animals, but only baseline data and one data point immediately following pMCAO were assessed. The purpose of this group was to determine the average flow values that would be collected in a normally flowing, healthy MCA and to examine the average initial effect of pMCAO on flow values compared to baseline.

**+0 h group** (Figure 1Q, n = 6) animals underwent pMCAO immediately followed by 2 hours of C2 whisker stimulation. These animals were then allowed to recover in their home cages and at 24 hours post-pMCAO, were re-anesthetized, imaged using LSI, and sacrificed so that TTC staining could be performed to assess cortical tissue health.

**+3 h group** (Figure 1R, n = 6) animals underwent the exact same protocol as the +0 h LSI animals except that there was a **3 hour delay** between pMCAO and whisker stimulation.

### Statistical Analysis

All plotting and statistics were performed using SYSTAT 11 (SYSTAT Software Inc., Chicago, IL, USA). Plots of means and standard error bars were provided for easy comparison between baseline and 24 hours post-pMCAO.

#### ISOI

Because there were no data to quantify when an infarct was sustained after pMCAO, post-pMCAO imaging evoked area and amplitude were converted to difference score values (post-occlusion - baseline), with values away from 0 signifying a change from baseline. A constant was added to difference values, which were then transformed with a natural log function to better satisfy the assumptions of an ANOVA (normal distribution for each subgroup of values, homogeneity of variance across means, independence between variance and means) and inferential statistics were performed on the transformed data. Following the ANOVA, specific contrasts were performed to identify which 24-hour whisker functional representations were significantly different from baseline. Separate ANOVAs followed by respective contrasts were performed for the three intrinsic signal phases of the ipsi-ischemic C2 representation, as well as for the initial dip signal phase of the ipsi-ischemic B1 representation and the initial dip signal phase of the C2 representation contralateral to the pMCAO. Alpha level was set to 0.05 and Bonferroni adjustments applied to account for the number of contrasts performed (3 contrasts per signal phase per whisker type for an adjusted alpha value = 0.0166).

#### Behavior

For neurological scores, three Pearson's chi-square tests were performed to compare the distribution of values for: the +3 h group versus controls, +3 h group versus +0 h group, and the +0 h group versus controls. The alpha level was set to 0.05 and Bonferroni adjustment applied to account for the three tests performed (0.05/3 = 0.017).

For both whisker guided and forepaw guided exploration, mean asymmetry scores were calculated and compared between groups. For each task, a separate repeated measures ANOVA was performed with three specific contrasts. The alpha level was set to 0.05 and Bonferroni adjustment applied to account for the three contrasts performed (0.05/3 = 0.017). The three contrasts were performed to identify any significant differences between either the +3 h animals asymmetry scores versus controls, +3 h versus +0 h animals, or +0 h animals asymmetry scores versus controls.

## Supporting Information

Table S1 Triphasic Details. All three ISOI signal phases (initial dip, overshoot, and undershoot) of the ipsi-ischemic whisker functional representation follow the same trends. Details regarding the area and amplitude of the initial dip, overshoot, and undershoot phases at baseline and 24 hours post-pMCAO. Group mean averages (±SEM) and post-hoc analysis are presented. * indicates a significant difference in either area or amplitude between baseline and 24 hour time points, alpha level was set equal to 0.0167 (0.05/3 contrasts per signal phase).
(0.16 MB TIF)

Figure S1 Thresholds of ISOI Analysis. +3h animals' loss of cortical function was verified at multiple levels of analysis. Left: The evoked whisker functional representation may be rendered three-dimensionally by plotting fractional change along the z-axis and its two-dimensional areal extent may be visualized and quantified at incrementally higher thresholds. The two thresholds used in this study are: 2.5×10^−4^ fractional change (FC; used to quantify area of activity) and 0.5×10^−4^ FC (used to determine presence of whisker functional representations). Right: A +3h subject's initial dip is visualized using both thresholds before and after pMCAO. Note the absence of activity at both thresholds after occlusion. The dark and light streaks are blood vessels.
(0.14 MB TIF)

Figure S2 +1h and +2h groups. Whisker stimulation can be protective if administered one or two hours post-pMCAO. A: In each plot, group baseline is paired with 24 hour data. Means and standard errors are provided for the area (first row) and amplitude (second row) of the ipsi-ischemic C2 whisker functional representation before and 24 hours after pMCAO. A value of zero indicates no response to whisker stimulation. Two +2h experimental group subjects (plotted individually in grey) showed a reduced area and amplitude 24 hours post-pMCAO (These same individuals also sustained a small infarct, depicted in B.). B: Location and extent of infarct present in the +1h and +2h experimental groups according to TTC analysis. Dark blue indicates area of infarct common to all subjects, and medium blue indicates maximum observed infarct area. No evidence of infarction was present in the +1h experimental subjects while two subjects in the +2h group sustained a small infarct. Atlas legend includes six vertical lines which correspond to each coronal section at 2 mm intervals from bregma. Brain map adapted from Paxinos 1998 [52].
(0.21 MB TIF)

Figure S3 TriPhasic Quantification. C2 whisker stimulation delivered within 1 hour (and possibly 2 hours) of pMCAO protects the whisker functional representations of both ipsi-ischemic C2 and B1 (a non-stimulated whisker). Contra-ischemic C2 representation was unaffected by pMCAO in all animals. In each plot, group baseline (left) is paired with 24 hour data (right). The horizontal hatched line demarcates zero, or no response to whisker stimulation. * indicates significant differences between baseline and 24 hour values. Shaded background regions highlight results that illustrate loss of whisker cortical function as a result of ischemic damage. A: Mean (±SE) area (left) and amplitude (right) of the initial dip (same as in Figure 2), overshoot, and undershoot of the ipsi-ischemic whisker C2 functional representation. B: Mean (±SE) area (left) and amplitude (right) of the ipsi-ischemic whisker B1 functional representation. C: Mean (±SE) area and amplitude of the contra-ischemic whisker C2 functional representation. Summary. Within subjects, the overall findings for the overshoot (A, second row) and the undershoot (A, third row) phases of the ipsi-ischemic C2 representation suggest that they behave similarly to the initial dip (A, first row). The same overall results as obtained for ipsi-ischemic whisker C2 were also obtained for another ipsi-ischemic whisker, B1. For contra-ischemic C2 whisker representation, however, no differences were observed between baseline and 24 hours after pMCAO for any animals in the three groups suggesting that the described differences observed between groups were constrained to the ischemic hemisphere, at least at 24 hours post-pMCAO.
(0.21 MB TIF)
