# Supplementary figures and images for: Mild Sensory Stimulation Completely Protects the Adult Rodent Cortex from Ischemic Stroke
Source: PLoS One. 2010 Jun 23;5(6):e11270. doi: 10.1371/journal.pone.0011270 (PMC2890583; doi:10.1371/journal.pone.0011270)

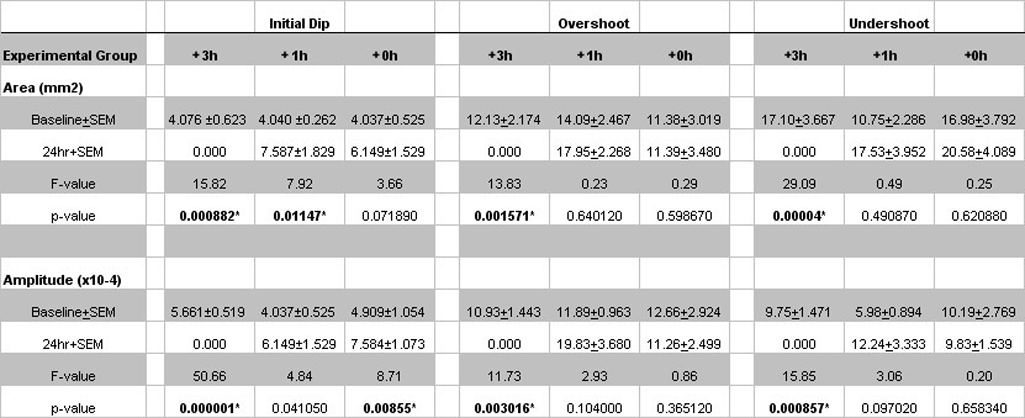

Supplement: Table S1 — Triphasic Details. All three ISOI signal phases (initial dip, overshoot, and undershoot) of the ipsi-ischemic whisker functional representation follow the same trends. Details regarding the area and amplitude of the initial dip, overshoot, and undershoot phases at baseline and 24 hours post-pMCAO. Group mean averages (±SEM) and post-hoc analysis are presented. * indicates a significant difference in either area or amplitude between baseline and 24 hour time points, alpha level was set equal to 0.0167 (0.05/3 contrasts per signal phase). (0.16 MB TIF) [file pone.0011270.s001.tif]

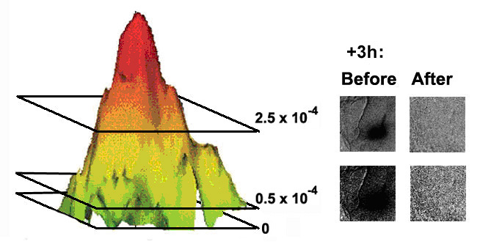

Supplement: Figure S1 — Thresholds of ISOI Analysis. +3h animals' loss of cortical function was verified at multiple levels of analysis. Left: The evoked whisker functional representation may be rendered three-dimensionally by plotting fractional change along the z-axis and its two-dimensional areal extent may be visualized and quantified at incrementally higher thresholds. The two thresholds used in this study are: 2.5×10^−4^ fractional change (FC; used to quantify area of activity) and 0.5×10^−4^ FC (used to determine presence of whisker functional representations). Right: A +3h subject's initial dip is visualized using both thresholds before and after pMCAO. Note the absence of activity at both thresholds after occlusion. The dark and light streaks are blood vessels. (0.14 MB TIF) [file pone.0011270.s002.tif]

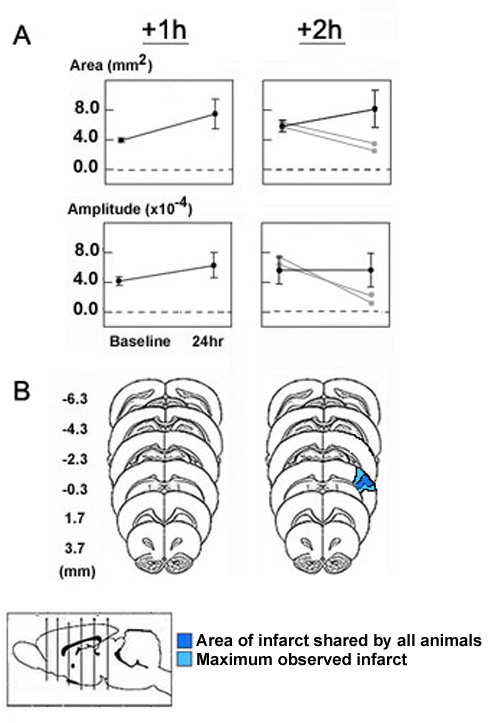

Supplement: Figure S2 — +1h and +2h groups. Whisker stimulation can be protective if administered one or two hours post-pMCAO. A: In each plot, group baseline is paired with 24 hour data. Means and standard errors are provided for the area (first row) and amplitude (second row) of the ipsi-ischemic C2 whisker functional representation before and 24 hours after pMCAO. A value of zero indicates no response to whisker stimulation. Two +2h experimental group subjects (plotted individually in grey) showed a reduced area and amplitude 24 hours post-pMCAO (These same individuals also sustained a small infarct, depicted in B.). B: Location and extent of infarct present in the +1h and +2h experimental groups according to TTC analysis. Dark blue indicates area of infarct common to all subjects, and medium blue indicates maximum observed infarct area. No evidence of infarction was present in the +1h experimental subjects while two subjects in the +2h group sustained a small infarct. Atlas legend includes six vertical lines which correspond to each coronal section at 2 mm intervals from bregma. Brain map adapted from Paxinos 1998 [52]. (0.21 MB TIF) [file pone.0011270.s003.tif]

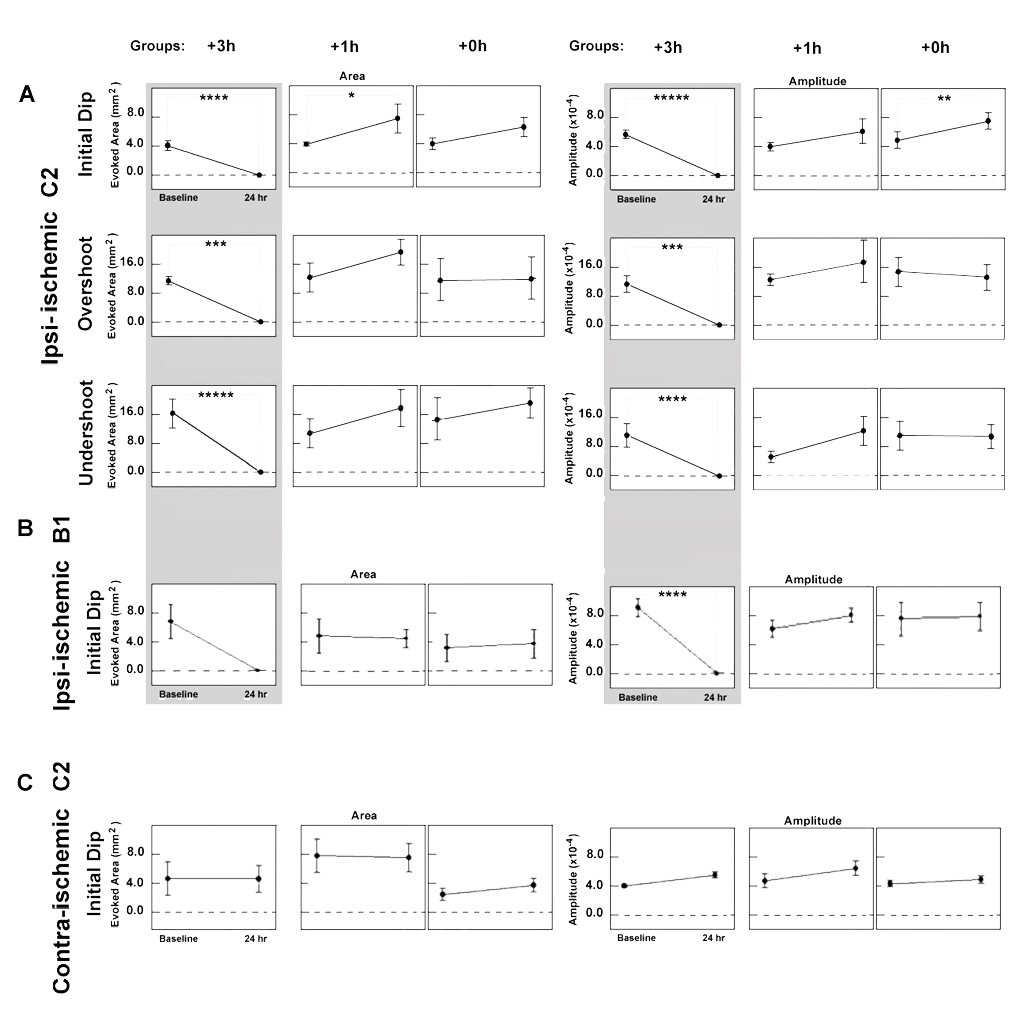

Supplement: Figure S3 — TriPhasic Quantification. C2 whisker stimulation delivered within 1 hour (and possibly 2 hours) of pMCAO protects the whisker functional representations of both ipsi-ischemic C2 and B1 (a non-stimulated whisker). Contra-ischemic C2 representation was unaffected by pMCAO in all animals. In each plot, group baseline (left) is paired with 24 hour data (right). The horizontal hatched line demarcates zero, or no response to whisker stimulation. * indicates significant differences between baseline and 24 hour values. Shaded background regions highlight results that illustrate loss of whisker cortical function as a result of ischemic damage. A: Mean (±SE) area (left) and amplitude (right) of the initial dip (same as in Figure 2), overshoot, and undershoot of the ipsi-ischemic whisker C2 functional representation. B: Mean (±SE) area (left) and amplitude (right) of the ipsi-ischemic whisker B1 functional representation. C: Mean (±SE) area and amplitude of the contra-ischemic whisker C2 functional representation. Summary. Within subjects, the overall findings for the overshoot (A, second row) and the undershoot (A, third row) phases of the ipsi-ischemic C2 representation suggest that they behave similarly to the initial dip (A, first row). The same overall results as obtained for ipsi-ischemic whisker C2 were also obtained for another ipsi-ischemic whisker, B1. For contra-ischemic C2 whisker representation, however, no differences were observed between baseline and 24 hours after pMCAO for any animals in the three groups suggesting that the described differences observed between groups were constrained to the ischemic hemisphere, at least at 24 hours post-pMCAO. (0.21 MB TIF) [file pone.0011270.s004.tif]
